# DAPT Attenuates Cadmium-Induced Toxicity in Mice by Inhibiting Inflammation and the Notch/HES-1 Signaling Axis

**DOI:** 10.3389/fphar.2022.902796

**Published:** 2022-04-29

**Authors:** Jia-Ying Yang, Dan-Yang Shen, Jun Wang, Jing-Feng Dai, Xiao-Yan Qin, Yang Hu, Rongfeng Lan

**Affiliations:** ^1^ Key Laboratory of Ecology and Environment in Minority Areas National Ethnic Affairs Commission, Center for Translational Neuroscience, College of Life and Environmental Sciences, Minzu University of China, Beijing, China; ^2^ Department of Cell Biology and Medical Genetics, School of Basic Medical Sciences, Shenzhen University Health Science Center, Shenzhen, China

**Keywords:** GFAP, γ-secretase, HES-1, IBA1, MAP2

## Abstract

The small molecule DAPT inhibits the Notch signaling pathway by blocking γ-secretase mediated Notch cleavage. Given the critical role of the Notch signaling axis in inflammation, we asked whether DAPT could block Notch-mediated inflammation and thus exert neuronal protection. We established a mouse model of chronic exposure to cadmium (Cd)-induced toxicity and treated it with DAPT. DAPT was effective in ameliorating Cd-induced multi-organ damage and cognitive impairment in mice, as DAPT restored abnormal performance in the Y-maze, forced swimming and Morris water maze (MWM) tests. DAPT also reversed Cd-induced neuronal loss and glial cell activation to normal as observed by immunofluorescence and immunohistochemistry of brain tissue sections. In addition, Cd-intoxicated mice showed significantly increased levels of the Notch/HES-1 signaling axis and NF-κB, as well as decreased levels of the inflammatory inhibitors C/EBPβ and COP1. However, DAPT down regulated the elevated Notch/HES-1 signaling axis to normal, eliminating inflammation and thus protecting the nervous system. Thus, DAPT effectively eliminated the neurotoxicity of Cd, and blocking γ-secretase as well as Notch signaling axis may be a potential target for the development of neuronal protective drugs.

## Introduction

Gamma-secretase is a highly conserved membrane-embedded protease with activity in cleaving the intracellular structural domains of receptors (Lu et al., 2014). In particular, there is a special interest in γ-secretase because of its key role in the pathogenesis of Alzheimer’s disease (AD) and cancer. The amyloid precursor protein (APP) of AD is cleaved by β-secretase to a secreted derivative, sAPP-β, and a 99-residue membrane-bound fragment, CTF-β, which is then cleaved by γ-secretase into CTF-γ and even pathogenic β-amyloid (Aβ) proteins, such as Aβ 40 and Aβ 42 (Kimberly and Wolfe, 2003; Marlow et al., 2003). In addition, Notch is another important target of γ-secretase (Kopan and Ilagan, 2009). γ-secretase exerts proteolytic activity that is required to liberate the intracellular structural domain of Notch and therefore plays a key role in the Notch signaling axis. Aberrant cleavage of APP and Notch has been reported to be highly associate with AD and cancer (Lopez-Nieva et al., 2021; Yang et al., 2021). Thus, it is a potential drug-target for the treatment of AD or anti-cancer drugs. γ-secretase inhibitors (GSIs) are thought to inhibit their cleavage of APP, thereby reducing the production of toxic Aβ (Dovey et al., 2001). Moreover, GSIs have been experimentally demonstrated to have anti-cancer activity in several types of tumor cells by inhibiting the activity of Notch signaling axis (Han and Shen, 2012; Peng et al., 2020; Liu et al., 2021).

In previous studies, we reported multi-organ damage and cognitive dysfunction in mice chronically exposed to cadmium (Cd) (Fan et al., 2021; Ren et al., 2021). Increased levels of Cd in the brain, as well as loss of neurons and activation of glial cells and the resulting oxidative stress and inflammation were found in Cd-intoxicated mice, (Wang and Du, 2013; Branca et al., 2019; Branca et al., 2020). With this in mind, we subsequently treated mice with Cd to induce cognitive impairment and demonstrated that antioxidants such as Edaravone were able to eliminate the toxicity of Cd, thereby protecting organs and the central nervous system (CNS) from the toxic damages of Cd (Fan et al., 2021). As Cd-induced neurotoxicity triggers significant inflammation in the brain, inflammatory signaling pathways, such as the Notch/HES-1 signaling axis and the RIP1-driven necroptosis pathways were found to be significantly activated (Ofengeim and Yuan, 2013; Ren et al., 2021). In light of this, in the current study, we considered whether inhibition of the Notch/HES-1 signaling axis could inhibit inflammation in the brain and thus attenuate Cd-induced neurotoxicity. Among the known compounds, DAPT (N-[N-(3, 5-difluorophenylacetyl)-l-propanoyl]-s-phenylglycine butyl ester) is an inhibitor of γ-secretase that indirectly inhibits the activity of the Notch signaling pathway (Dovey et al., 2001). Therefore, we tested the neuroprotective effect of DAPT to see if it could scavenge Cd toxicity by inhibiting the Notch/HES-1 signaling axis.

## Materials and Methods

### Animal and Ethical Statement

ICR mice (6-8 weeks) were obtained from Beijing Vital River Laboratory Animal Technology Co., Ltd., under license No. SYXK-2017-0005. Mice were housed under a 12 h/12 h light/dark cycle at a temperature of 22-24°C and 60 ± 10% humidity, with ad libitum access to water and food. The animal study was reviewed and approved by the Animal Care and Use Committee of the Minzu University of China.

### Drug Treatment

ICR mice were randomly divided into 4 groups (*n* = 10) including Control, Cd (5 mg/kg of CdCl_2_), Cd+DAPT (CdCl_2_ 5 mg/kg + DAPT 50 mg/kg), and DAPT (50 mg/kg). CdCl_2_ was injected subcutaneously every other day for 28 days. DAPT was administered intragastrically once daily for 28 days. In addition, for the control group, intragastric administration was also performed with saline. CdCl_2_ (CAS #10108-64-2) was provided by Macklin Inc. Shanghai, China. DAPT (CAS #208255-80-5) was purchased from Selleck Chemicals LLC, China.

### Behavioral Procedures

The cognitive function of mice was tested of by the MWM test, as previously described (Ren et al., 2021). Briefly, in the acquired training, mice were placed in a large circular pool (110 cm in diameter) filled with opaque water (25°C) and allowed to escape to a hidden platform submerged 1 cm below the water surface, the location of which could only be identified by spatial memory. The training lasted for 6 days, during which the escape latency was shortened due to learning memory and the direct swimming routes of the mice. After the end of the training, the platform was removed and the probe trail was performed. In the above experiments, a video-image system and behavioral tracking software (WMT-100S, Chengdu Coman Software Co., Ltd.) was used to record the residence time of the mice in the target quadrant where the platform was initially placed, the number of times the mice crossed the area where the platform was located, and the trajectory of the mice.

The Y-maze spontaneous alternation experiment was used to test the willingness of animals to explore new environments (Kraeuter et al., 2019; Ren et al., 2021). The maze consisted of three equal Y-shaped arms (30 cm long, 6 cm wide and 15 cm high) with an angle of 120° between them. Animals were allowed to explore freely from the top of one arm of the maze. The animal was considered to have entered the maze when all four of its limbs were inside the arms. Correct spontaneous alternation occurred when the entering arm was different from the first two arms. The order in which the animal entered each arm over a 10-min period was recorded. Finally, the total alternation times and the number of spontaneous alternations were calculated. Alternations (%) = (number of spontaneous alternations/number of arm entries-2) × 100.

A forced swimming test (FST) was performed to assess the depression-like phenotype of the animals (Yan et al., 2010). The experiment was performed in a transparent plastic cylindrical water tank (diameter 10 cm, height 38 cm). Water at 25°C was poured into the tank (height 25 cm), the mice were gently placed above the water surface and then released for swimming for a 6-min swim. The first minute was set as an adaption and was not recorded. The next 5 min were recorded by Taimeng FST-100 software. The immobility time of mice within these 5 min was calculated.

### Immunofluorescence

Brain sections were fixed in 4% paraformaldehyde for 15 min at room temperature, rinsed three times with PBS, incubated with 0.2% Triton X-100 for 10 min, and then blocked with 10% goat serum in PBS for 1 h and immuno-labeled with antibodies specific for microtubule-associated protein 2 (MAP2) (#17490-1-AP), GFAP (#16825-1-AP) or IBA1 (#10904-1-AP) overnight at 4°C, and then incubated with Alexa Fluor 488- or 596- conjugated goat anti-rabbit IgG (H+L) (#ZF-0511, ZSBG-Bio). Nuclei were stained with DAPI (4′, 6-diamidino-2-phenylindole). Images were taken using a Leica TCS SP8 confocal microscope.

### Western Blotting

Western blotting was performed as previously described (Li et al., 2021). Briefly, 50 μg of proteins extracted from the hippocampus or prefrontal cortex was loaded onto SDS-PAGE and separated, then transferred to a nitrocellulose membrane. After blocking the nonspecific binding sites with skim milk, proteins were detected with the following antibodies. Antibodies specific for RBP-Jκ (#14613-1-AP, dilution 1:1,000) were provided by Proteintech, Inc. NF-κB p65 (#BD-PT5770, dilution 1:1,000) was provided by Biodragon. Notch1 (#AF5249, dilution 1:1,000), phospho-NF-κB p65 (Ser536) (#AF5881, dilution 1:1,000) and HES-1 (#AF2167, dilution 1:1,000) were purchased from Beyotime Biotechnology. COP1 (#bs-13925R, 1:1,000) antibody was purchased from Bioss Antibodies. β-actin antibody (13E5) (#4970S, 1:1,000) was purchased from Cell Signaling Technologies. Western blotting results were scanned by Odyssey CLx infrared fluorescence imaging system (LI-COR Biosciences). The relative expression levels of proteins were normalized according to their optical density and quantified in ImageJ software.

### H & E Staining

In brief, after de-paraffinization and rehydration, sections were stained with Harris modified hematoxylin solution for 5 min, followed by 5 immersions in 0.5% acidic ethanol (0.5% HCl in 70% ethanol), following by rinsing with distilled water, staining with eosin solution for 1 min, and then gradual dehydration in alcohol and in xylene for 30 s. The mounted slides were examined using an upright microscope (BX53, Olympus, Japan).

### Immunohistochemical Staining

IHC staining of brain sections was performed for histological analysis by Wuhan Servicebio Technology Co. Ltd. Briefly, brain tissue was dissected from the prefrontal cortex and hippocampus, and paraffin embedded, sectioned, antigens retrieved, and finally immunostained with antibodies. Antibodies specific for GFAP (#16825-1-AP), IBA1 (#10904-1-AP) and TNF-α (#bsm-33207M) were provided by Proteintech Inc. or Bioss, respectively, to indicate neurons, astrocytes, microglia and inflammation. Results were examined and processed with Image Pro Plus 6.0 software (Media Cybernetics, Inc.).

### Data and Statistics

All data are expressed as mean ± s.e.m. Analysis of variance (ANOVA) and Tukey’s multiple tests were performed by GraphPad Prism software to determine statistical significance between groups and were plotted, as well as illustrated in the figure legends. The Mann Whitney’s test was performed for non-parametric analysis.

## Results

### DAPT Attenuates Cd-Induced Multi-Organ Damage and Cognitive Impairment in Mice

**FIGURE 1:**
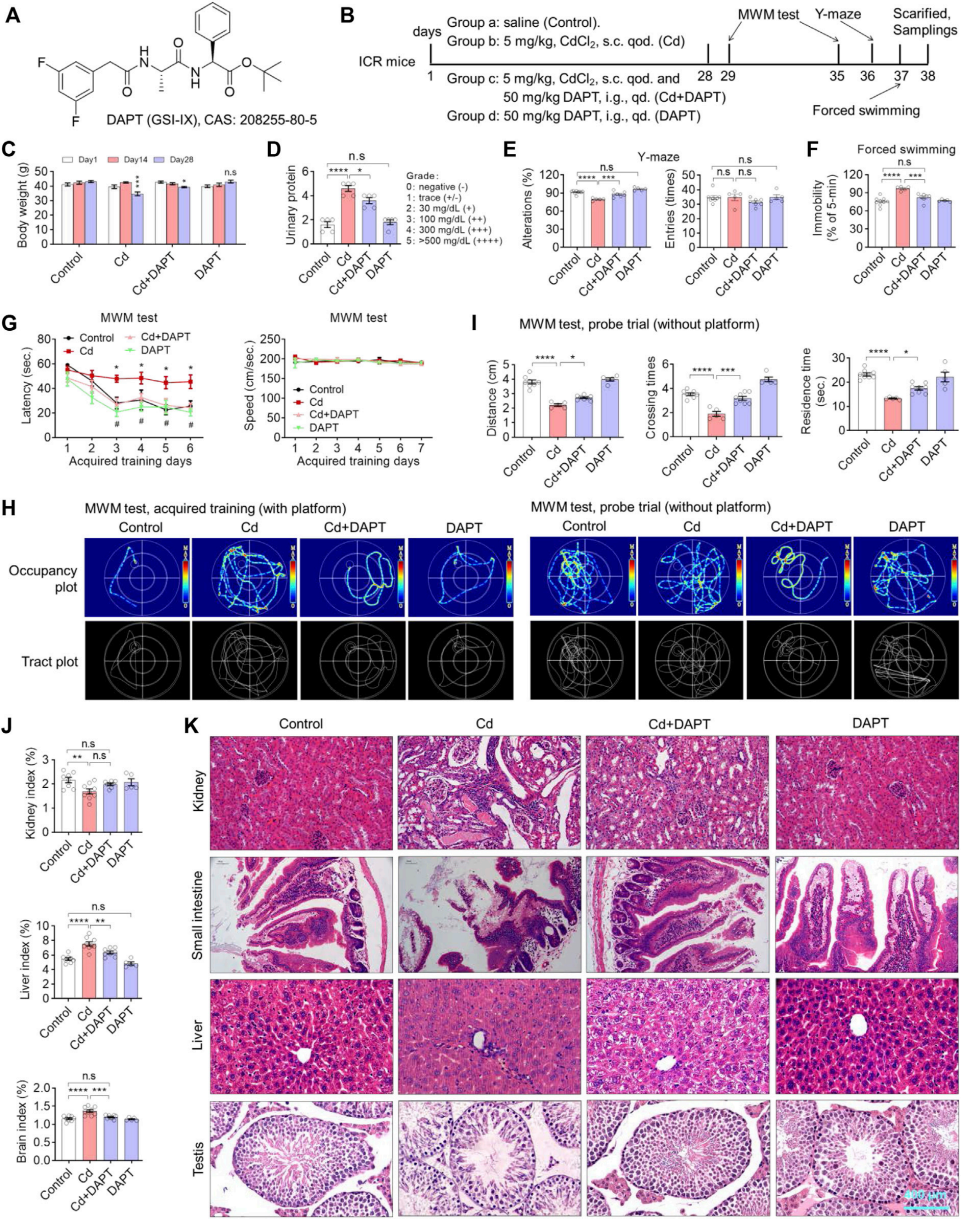
DAPT attenuates Cd-induced multi-organic damage and cognitive impairment in ICR mice. **(A)** Chemical structure of DAPT, a γ-secretase inhibitor IX. **(B)** Schedule of animal experiments. s.c. qod., subcutaneous injection, every other day. i.g. qd., intragastric administration, once daily. **(C)** DAPT prevented the Cd-induced body weight loss in mice. **(D)** DAPT attenuated the Cd-induced elevation of urinary proteins. **(E)** DAPT rescued the decrease of spontaneous alterations induced by Cd as tested in the Y-maze. **(F)** DAPT improved the performance of mice in forced swimming test. **(G)** Escape latency of ICR mice in the acquired training (with platform) in the MWM test. **(H)** Representative occupancy or tract plots of mice in the MWM test. **(I)** Probe trails in the MWM test showing the residence time and distance moved in the target quadrant, as well as the number of crossing times in the area where the platform was originally placed. **(J)** Organ indices of liver, kidney and brain. Cd-induced toxicity caused renal atrophy and hepatic or cerebral edema according to organ/body mass index. **(K)** H & E staining showing that Cd-induced tissue damage was ameliorated by DAPT treatment. Scale bar, 400 μm. In this figure, in C, E, F, G and J, one-way ANOVA, post hoc Tukey’s test was performed. In D, the Mann-Whitney *U*-test for non-parametric analysis was performed. In H, two-way RM ANOVA, post hoc Tukey’s test was performed. n.s, not significant; *p < 0.05; **p < 0.01; ***p < 0.001; ***p < 0.0001 (Cd vs. Control); #*p* < 0.05 (Cd + DAPT vs. Cd).

We first established a chronic Cd-exposured ICR mouse model by administering 5 mg/kg of CdCl_2_ every other day for 4 weeks. Accordingly, a DAPT treatment group (Cd+DAPT) and a separate DAPT group were set up (Figures 1A,B). During the experiment, body weight and urine protein were measured to track the damage of Cd on the organs of the mice (Figures 1C,D). After drug treatment, behavioral tests were performed on the mice, including the MWM test, Y maze and forced swimming test. The results showed that the body weight of mice changed significantly after 4 weeks of Cd treatment, i.e. the body weight of Cd-treated mice was significantly lower on day 28 than on day 1 (Figure 1C, Cd, *p* < 0.001), whereas the body weight of control mice increased naturally with the increase in weeks of age. Mice treated with DAPT recovered their body weight compared to the Cd group, and their difference was significantly reduced compared with day 1 (Figure 1C, Cd + DAPT, *p* < 0.05). Correspondingly, urinary protein increased in mice treated with Cd, with a significant difference compared to the control group (Figure 1D, *p* < 0.0001), while urinary protein decreased in mice treated with DAPT, with a difference compared to the Cd group (Figure 1D, *p* < 0.05), while there was no change in mice treated with DAPT alone (Figure 1D, n.s).

As confirmed by the Y-maze test, the environmental exploration and memory abilities of Cd-poisoned mice were significantly reduced, whereas the performance of mice in the DAPT-treated group recovered significantly, and their environmental exploration abilities were similar to those of the control group (Figure 1E). Correspondingly, in the forced swimming experiment, the resting time was significantly increased in the Cd-intoxicated mice (Figure 1F, *p* < 0.0001), while in the DAPT-treated mice, the resting time was shortened and did not differ from the control group (Figure 1F, Cd+DAPT, not significant). In the MWM experiment, after 6 days of training, mice took significantly less time to find the platform and showed learning memory (Figure 1G, control). Then, Cd-treated mice then took significantly longer to find the platform than the control group, and the improvement was not significant as the number of training days increased (Figure 1G, Cd). In contrast, mice in the DAPT-treated group showed a significant improvement in learning and memory, i.e., regained the ability to find the underwater platform similar to control mice (Figure 1G, Cd+DAPT). In this experiment, there was no difference in the swimming speed of the mice in the water maze. Corresponding to these results, the swimming path of mice recorded by thermal infrared imaging also reflected changes in their learning and memory ability (Figure 1H). On this basis, at day 7 of the experiment, the platform was removed and the mice were allowed to perform a 1-min swimming test (probe trail). We found that mice in the control and DAPT-treated groups had more residence time, more times crossing the platform position and more swimming distance in the area where the platform was originally placed compared to the Cd-treated group, i.e. the mice showed the ability to remember the location of the underwater platform. In contrast, Cd-intoxicated mice had a significant impaired ability to do so and showed no sense of purpose while swimming (Figures 1H,I). Consistently, organic indices (organ/body weight) also showed that damage caused by Cd exposure resulted in kidney atrophy, while the liver and brain showed enlargement or hyperplasia (Figure 1J). H&E staining also confirmed that Cd exposure caused significant damage to the kidney, liver, small intestine and testes, while the damage in mice treated with DAPT was effectively repaired (Figure 1K).

### DAPT Facilitates the Clearance of Cd and Avoids the Loss of Neurons in the Brain

**FIGURE 2:**
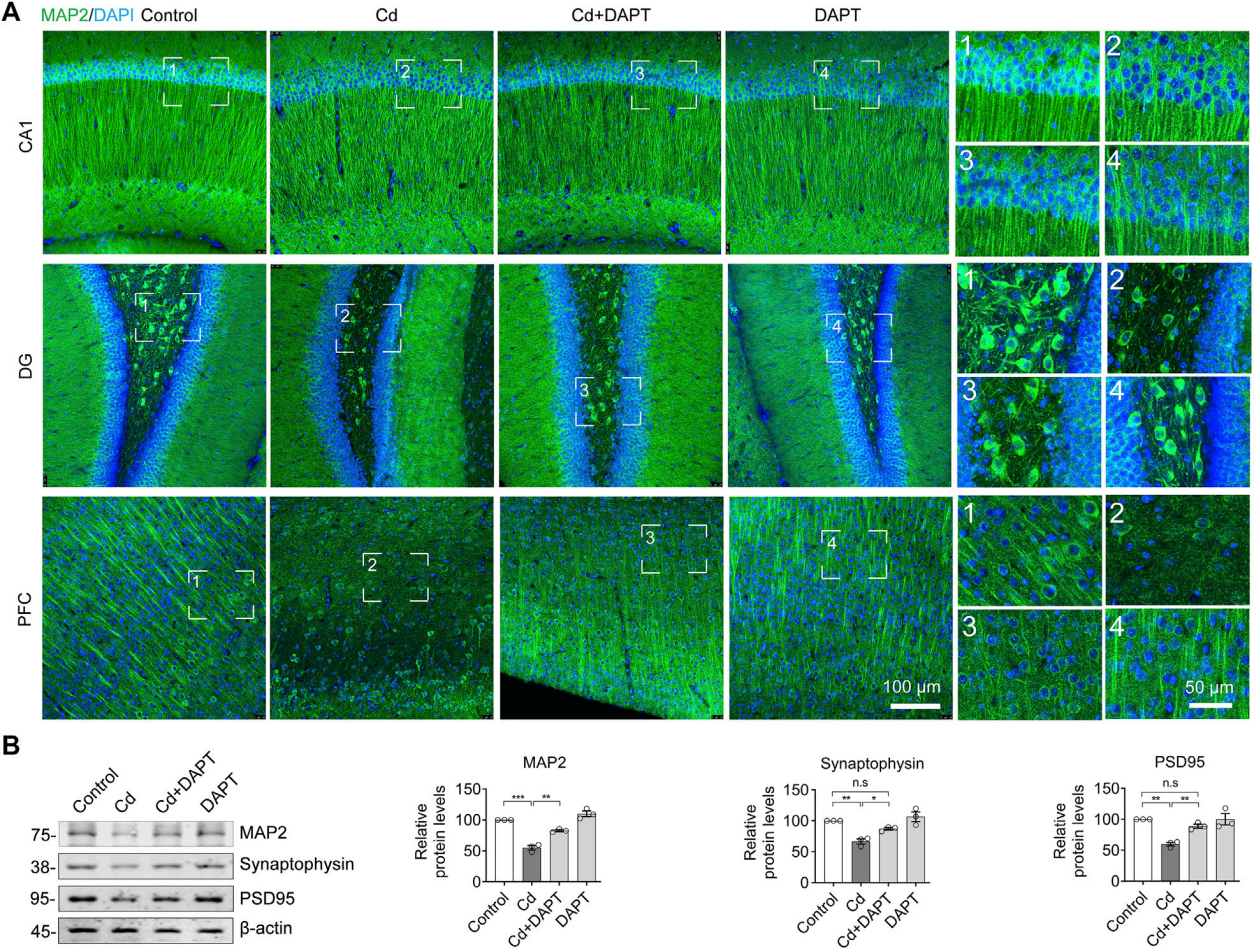
DAPT protects neurons from Cd-induced toxicity. **(A)** IF staining of neurons in the hippocampal regions CA, DG and in the prefrontal cortex (PFC) using an antibody specific for MAP2. **(B)** Western blotting detected the levels of MAP2 and the neuronal synaptic proteins Synaptophysin and PSD95 in the hippocampus. The intensity of the blotted bands was quantified using ImageJ software and normalized to β-actin. One-way ANOVA with post hoc Tukey’s test was performed. n.s, not significant; *p < 0.05; **p < 0.01; ***p < 0.001.

Brain tissue was surgically isolated from mice after animal experiments, sectioned and immunofluorescence stained with MAP2 antibody to identify neurons. The results showed that a significant reduction of neurons in the brains of Cd-intoxicated mice, such as in the CA1 and DG regions of the hippocampus, and in the prefrontal cortex (PFC) region (Figure 2A, Cd). After DAPT treatment, the number of neurons in these regions was restored. Correspondingly, MAP2 and synaptic-associated proteins such as Synaptophysin and PSD95 were reduced due to Cd toxicity but were restored after DAPT treatment (Figure 2B). Quantitative optical density analysis of the protein bands in the Western blot results also confirmed these changes. These results suggest that the clearance of Cd toxicity and recovery of neurons by DAPT treatment are consistent with the results of behavioral experiments previously observed in mice.

### DAPT Inhibits Cd Toxicity-Induced Glial Cell Activation

**FIGURE 3:**
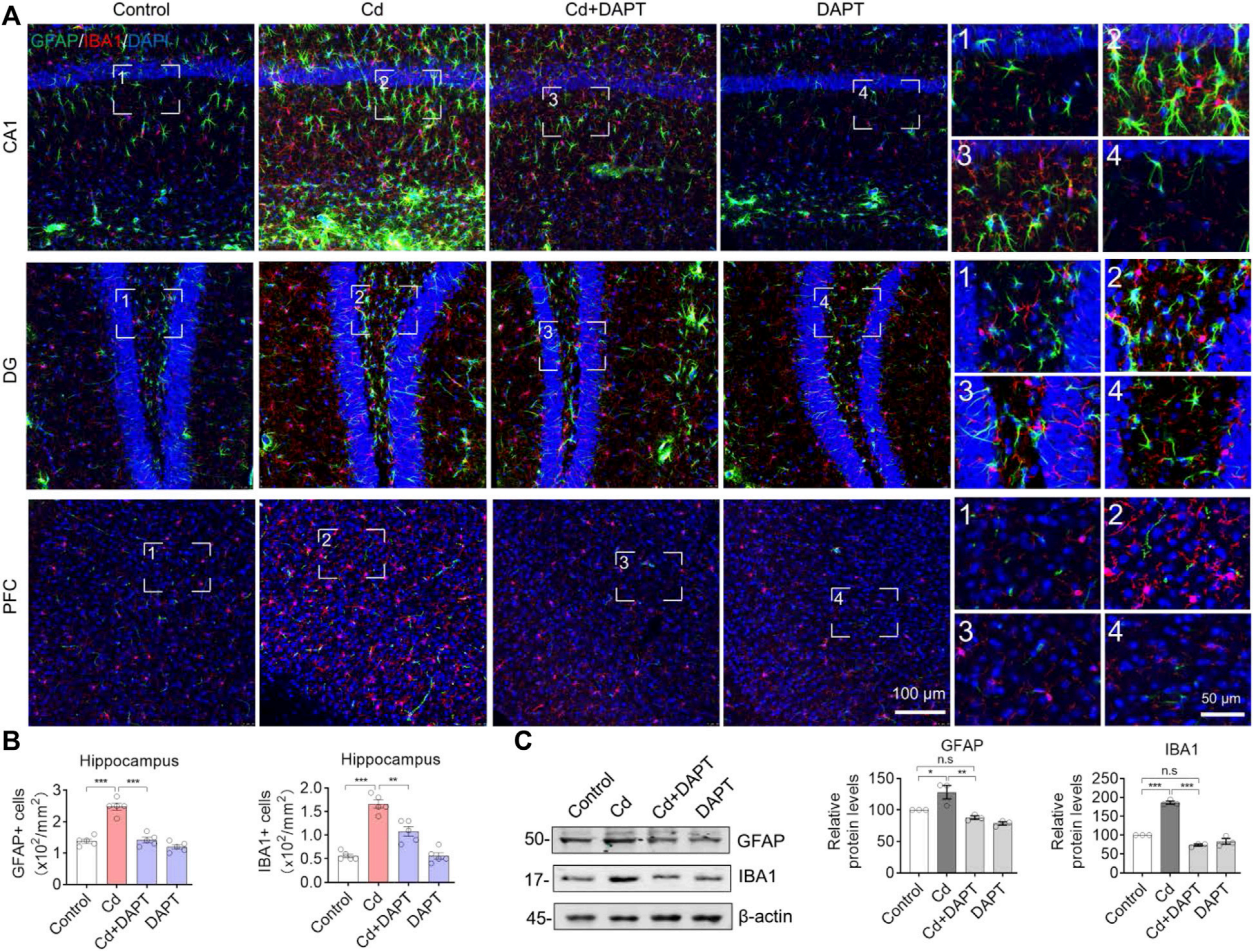
Representative IF images of GFAP and IBA1. **(A)** DAPT antagonized the activation of microglia and astrocytes induced by Cd-toxicity, as shown by IF staining of GFAP and IBA1 using specific antibodies, respectively. **(B)** GFAP+ or IBA1+ cells were manually counted in images of **(A)**. **(C)** Western blots analysis the protein levels of GFAP and IBA1. In B-C, one-way ANOVA and post hoc Tukey’s test was performed. n.s, not significant; *, *p* < 0.05; **, *p* < 0.01; ***, *p* < 0.001.

In the brains of Cd-intoxicated mice, we found increased GFAP+ and IBA1+ cells by staining for the characteristic proteins in microglia and astrocytes (Figure 3A, Cd), indicating that the glial cells were activated in the brains of chronically Cd-exposed mice. In contrast, the number of GFAP+ and IBA1+ cells was significantly lower in the brains of mice in the DAPT treated group (Cd+DAPT) and the DAPT alone treated group (Figure 3A). Statistically, there was a significant difference in the number of GFAP+ and IBA1+ cells between the above experimental groups (Figure 3B). In addition, protein assays of GFAP and IBA1 showed that the expression of GFAP and IBA1 increased in Cd-intoxicated mice, while the levels of these two proteins returned to normal after DAPT treatment (Figure 3C, Cd+DAPT).

**FIGURE 4:**
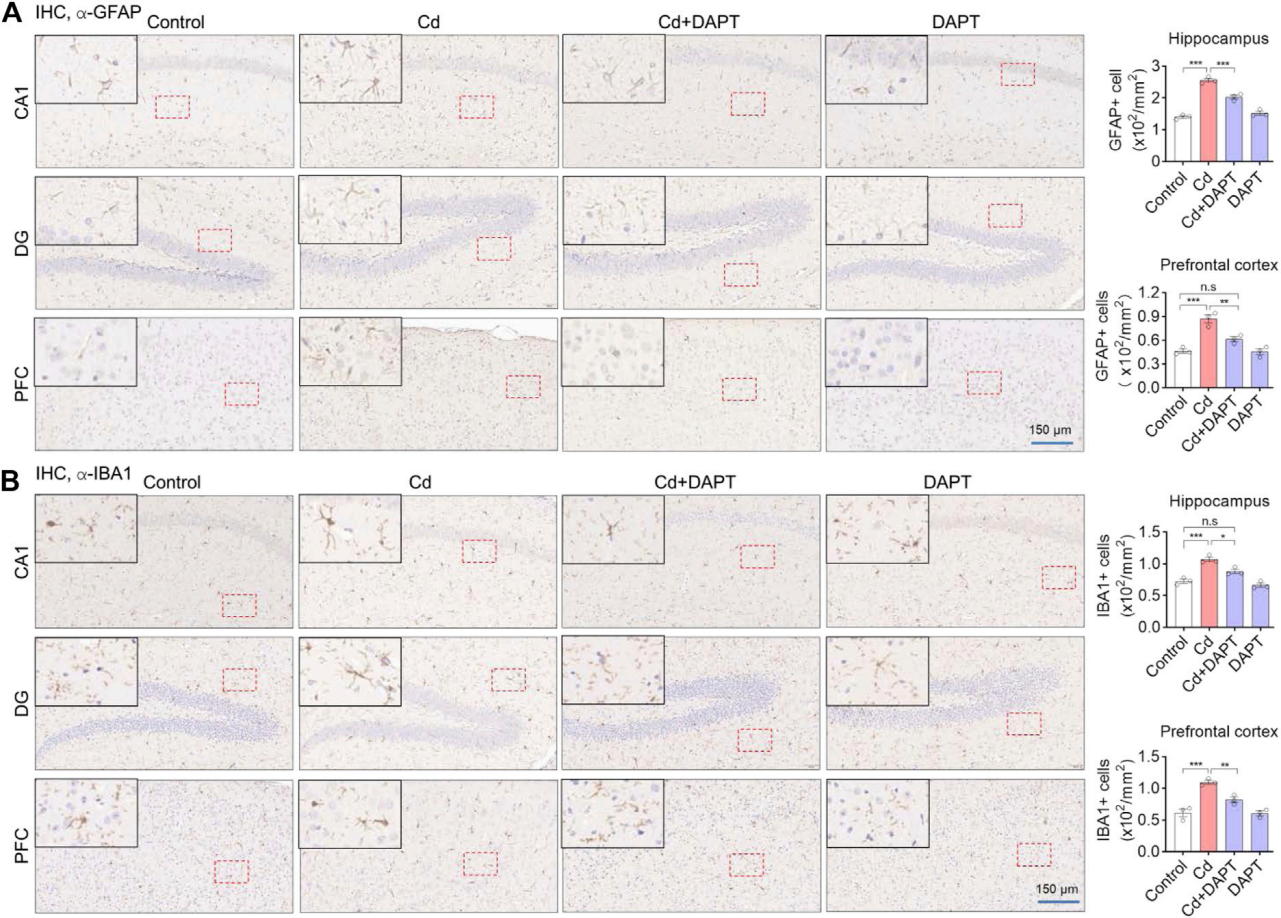
IHC staining of GFAP and IBA1 in the prefrontal cortex (PFC) and hippocampal regions CA1 and DG. **(A,B)** Representative IHC images showing Cd-induced activation of astrocytes and microglia, such as an increase in the number of GFAP+ and IBA1+ cells, but this can be restored by DAPT treatment. GFAP+ and IBA+ cells was manually counted and divided by the corresponding area. One-way ANOVA with post hoc Tukey’s test was performed. n.s, not significant; *p < 0.05; **p < 0.01; ***p < 0.001.

**FIGURE 5:**
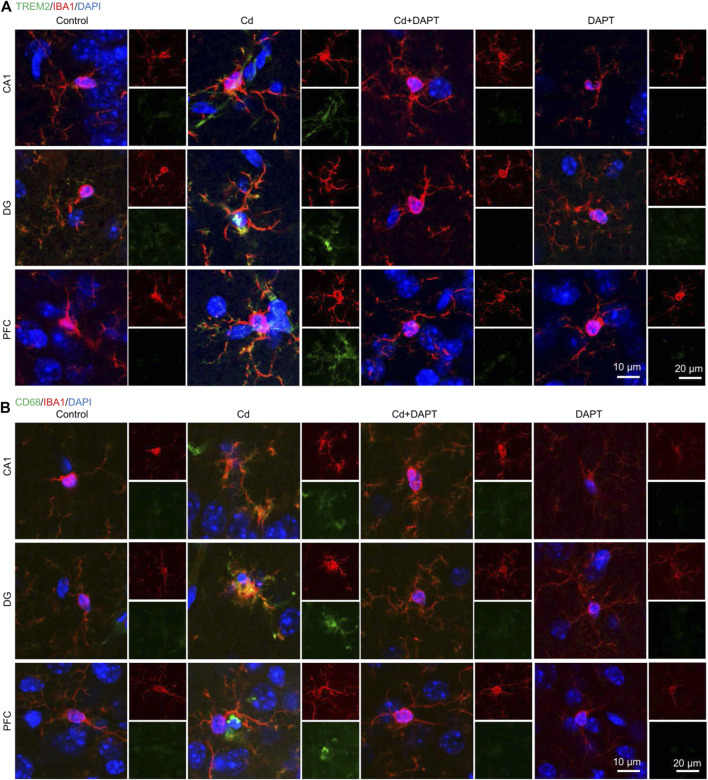
IF co-staining of TREM2 or CD68 with IBA1 in brain tissue sections. **(A)** Cd toxicity activated microglia, as evidenced by increased levels of TREM2 and CD68. DAPT attenuated Cd toxicity and microglia activation, and restored the CD68 upregulation. **(B)** Schematic diagram showing DAPT-mediated attenuation of Cd toxicity and restoration of homeostasis in the neuronal system.

In addition, brain sections from the hippocampus and prefrontal cortex (PFC) were used for IHC staining to label GFAP+ and IBA1+ cells. Very similar to the results of IF (Figure 3), we found that a significant increase in the number of GFAP+ and IBA1+ cells in Cd-intoxicated mice, and DAPT was able to eliminate this change and restore glial cells to a normal state (Figure 4). In addition, microglia can be activated by stress and the need to clear damaged cells, as indicated by elevated expression of triggering receptor expressed on myeloid cells 2 (TREM2) and macrosialin (CD68), suggesting their enhanced phagocytic capacity. TREM2 is a receptor for Aβ42 or damaged cells that mediate their uptake and degradation by microglia (Yeh et al., 2016; Zhao et al., 2018), while CD68 is a marker protein indicative of microglia phagocytic activity (Wong et al., 2005; Chistiakov et al., 2017). In view of this, immunofluorescence observations on microglia showed a significant increase in the expression of TREM2 and CD68, which was associated with microglia phagocytosis in response to Cd toxicity, possibly enhance cellular clearance of Cd (Figure 5). In contrast, after DAPT treatment, the levels of TREM2 and CD68 were down-regulated and returned to normal (Figure 5, Cd+DAPT). The above results consistently suggest that DAPT can effectively inhibit Cd-induced glial cell activation and eliminate neuronal inflammation.

### DAPT Inhibits the Notch/HES-1 Signaling Axis to Eliminate the Toxicity of Cd

**FIGURE 6:**
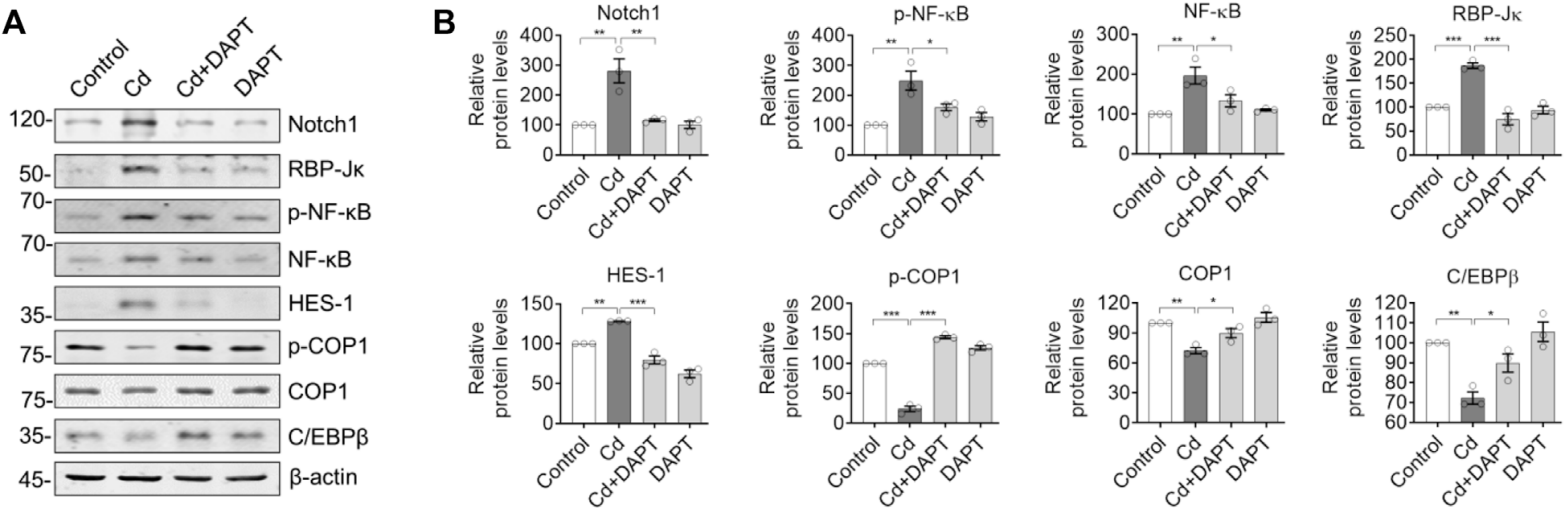
DAPT restores the Notch/HES-1 signaling axis to mitigate Cd-induced toxicity in mice. **(A)** Western blots of proteins in the Notch/HES-1 signaling axis. **(B)** Relative protein levels in **(A)**. One-way ANOVA with post hoc Tukey’s test was performed. *p < 0.05; **p < 0.01 or ***p < 0.001.

We examined the expression of Notch/HES-1 signaling pathway and its related proteins such as NF-κB. The results showed that the activity of Notch/HES-1 signaling axis was significantly enhanced in Cd-poisoned mice, i.e. Notch1, RBP-Jk, HES-1 and NF-kB were significantly increased, while its negative regulators such as constitutive photomorphogenesis protein 1 homolog (COP1) and CCAAT/enhancer-binding protein beta (C/EBPβ) were significantly decreased (Figure 6). This suggests that the activity of the Notch/HES-1 signaling axis is significantly enhanced in Cd-intoxicated mice. In contrast, Notch/HES-1 signaling axis activity was significantly down-regulated in the DAPT-treated mice (Figure 6, Cd+DAPT), i.e., the levels of Notch1, RBP-Jk, HES-1, NF-kB and other proteins were significantly reduced and returned to normal (compared with controls). At the same time, the protein levels of COP1 and C/EBPβ were restored (Figure 6, Cd+DAPT). These results suggest that DAPT, as an inhibitor of γ-secretase, significantly inhibits the activity of the Notch/HES-1 signaling axis, thereby protecting the nervous system.

## Discussion

In the present study, we established a mouse model of chronic Cd exposure and confirmed Cd-induced multi-organ toxicity and neurotoxicity. DAPT, an inhibitor of γ-secretase, was successfully used to eliminate Cd toxicity in the mouse model and its effectiveness against Cd toxicity was confirmed in biochemical, histological and animal behavioral experiments. In addition, we found that Cd induced an increase in the number of glial cells and the activation of the Notch/HES-1 signaling axis in the mouse brain. The expression levels of the Notch/HES-1 signaling axis and related factors were also ameliorated. These results indicate that DAPT can effectively alleviate Cd-induced neurotoxicity and exert neuroprotective activity.

Under normal conditions, Cd barely enters the brain and directly harms the CNS due to the presence of blood-brain barrier (BBB) (Wang and Du, 2013). However, Cd can still reach the basement membrane cells of the BBB through the blood circulation (Branca et al., 2020). Chronic exposure to Cd can damage these cells that make up the BBB, thereby disrupting the integrity of the BBB and allowing Cd to enter the CNS (Branca et al., 2019). Death receptor-mediated signaling pathways, such as those driven by RIP1, lead to neuronal death (Xu et al., 2018). Loss of neurons in brain regions associated with functions such as learning and memory will produce significant cognitive deficits, hence the significant cognitive impairment and other functional deficits observed in our previous studies in mice chronically exposed to Cd (Fan et al., 2021). Fortunately, clinically applied antioxidants such as Edaravone, as well as the natural product ginsenoside Rg1 are effective in alleviating these Cd-induced symptoms (Ren et al., 2021). Accordingly, we also observed a restoration of antioxidant capacity in the blood and brain, a reduction in inflammation and neuronal recovery. These results are very similar to the effects of DAPT in the current study, although the direct antioxidant and anti-inflammatory effects of DAPT have not been demonstrated, it did inhibit the Notch/HES-1-mediated inflammatory pathway (Figure 6) and we did observe tissue and organ damage, such as in the liver in Cd-intoxicated mouse model. In particular, in the CNS, we observed neuronal recovery and reduced inflammation in mice, resulting in improved cognitive function (Figures 1, 2). The above evidence suggests that we can use DAPT in mouse models in an attempt to counteract the toxicity of Cd or protect the organism from its deletrious effects. Furthermore, the effectiveness of DAPT in eliminating Cd toxicity suggests that suppressing excessive inflammation in the CNS and restoring neuronal activity is an effective neuroprotective strategy.

It is worthwhile to explore in depth how DAPT exerts its role in eliminating Cd toxicity. The first step may be to reduce the uptake of Cd by the CNS. DAPT has been reported to modulate the permeability of the BBB, affecting the movement of substances into and out of the CNS (Zhang et al., 2013), so it is of interest whether DAPT could reduce the influx of Cd into the brain via this pathway (Sun et al., 2019). Furthermore, by inhibiting the Notch/HES-1 signaling pathway, DAPT attenuates the inflammatory response in the CNS, which is important for reducing pathological inflammation-induced neuronal loss and may even modulate microglia functions, such as inhibiting their excessive inflammation and release of inflammatory factors, enhancing their phagocytosis, and facilitating the clearance of Cd and other harmful substances in the CNS. Because microglia have been shown to act as scavengers in the CNS, they are able to act as phagocytes to clear toxins or eliminate damaged cell bodies under the right conditions. However, the above possible mechanism remains a hypothesis to be tested in depth in subsequent experiments. Nevertheless, the neuroprotective effects of DAPT that we demonstrated in this study may have implications for the design and development of novel neuroprotective compounds or for the prevention and treatment of clinical disorders of the CNS.

In conclusion, DAPT attenuates Cd-induced multi-organ damage and cognitive dysfunction in mice and avoided the loss of neurons in the brain. And the above effects of DAPT are likely to be achieved by inhibiting Cd toxicity-induced glial cell activation and Notch/HES-1 signaling axis.

## Data Availability

The original contributions presented in the study are included in the article/Supplementary Material, further inquiries can be directed to the corresponding authors.
